# Spatially and Financially Explicit Population Viability Analysis of *Maculinea alcon* in The Netherlands

**DOI:** 10.1371/journal.pone.0038684

**Published:** 2012-06-14

**Authors:** Viktoriia Radchuk, Michiel F. WallisDeVries, Nicolas Schtickzelle

**Affiliations:** 1 Biodiversity Research Centre, Earth & Life Institute, Université catholique de Louvain, Louvain-la-Neuve, Belgium; 2 De Vlinderstichting/Dutch Butterfly Conservation, Wageningen, The Netherlands; 3 Laboratory of Entomology, Wageningen University, Wageningen, The Netherlands; Lund University, Sweden

## Abstract

**Background:**

The conservation of species structured in metapopulations involves an important dilemma of resource allocation: should investments be directed at restoring/enlarging habitat patches or increasing connectivity. This is still an open question for *Maculinea* species despite they are among the best studied and emblematic butterfly species, because none of the population dynamics models developed so far included dispersal.

**Methodology/Principal Findings:**

We developed the first spatially and financially explicit Population Viability Analysis model for *Maculinea alcon*, using field data from The Netherlands. Implemented using the RAMAS/GIS platform, the model incorporated both local (contest density dependence, environmental and demographic stochasticities), and regional population dynamics (dispersal rates between habitat patches). We selected four habitat patch networks, contrasting in several basic features (number of habitat patches, their quality, connectivity, and occupancy rate) to test how these features are affecting the ability to enhance population viability of four basic management options, designed to incur the same costs: habitat enlargement, habitat quality improvement, creation of new stepping stone habitat patches, and reintroduction of captive-reared butterflies. The PVA model was validated by the close match between its predictions and independent field observations on the patch occupancy pattern. The four patch networks differed in their sensitivity to model parameters, as well as in the ranking of management options. Overall, the best cost-effective option was enlargement of existing habitat patches, followed by either habitat quality improvement or creation of stepping stones depending on the network features. Reintroduction was predicted to generally be inefficient, except in one specific patch network.

**Conclusions/Significance:**

Our results underline the importance of spatial and regional aspects (dispersal and connectivity) in determining the impact of conservation actions, even for a species previously considered as sedentary. They also illustrate that failure to account for the cost of management scenarios can lead to very different conclusions.

## Introduction

The development of metapopulation theory [1] has contributed significantly to conservation practice for species with patchy distribution and limited dispersal by offering a framework for decision making. Efforts for conservation or restoration can be directed at improving connectivity, increasing habitat area, improving habitat quality or a combination of these [2]. Initially, much attention was devoted to the purely spatial aspects of metapopulation functioning [3]. Subsequently, more emphasis was put on aspects of habitat quality as a crucial factor in the conservation of more sedentary species [4], [5]. In the end, however, conservation practice requires tools to make a balanced decision between the various options. Population Viability Analysis (PVA) [6] has the potential to offer such tools but efforts to develop these for so-called “sedentary species” have been limited by poor information on dispersal. In this paper we explore the potential of PVA models to assist conservation efforts for the endangered butterfly *Maculinea alcon.*

Butterflies are often used as model species for conservation biology studies in our changing world [7], [8]. Among them, the species of the *Maculinea* genus (Lepidoptera, Lycaenidae) are of special interest. All five *Maculinea* species are listed as “Endangered” or “Vulnerable” in Europe [9]. But more importantly, they are obligate myrmecophiles: the life cycle contains a phase spent within an ant nest [10]. This interaction is species specific, each *Maculinea* species being associated to a certain ant species [11], [12]; some regional variation in these associations has been observed, however [13]. In conservation biology, the complicated life history of *Maculinea* butterflies has made them fruitful models for the novel field of preserving species interactions [14].

Given this special interest, scientists put a lot of effort in the investigation of the *Maculinea* life history, through field studies [15], [16], [17], [18], lab experiments [19], [20], [21], and also modelling [22], [23], [24], [25]. Models can indeed be a good (and sometimes the only) option to study specific aspects of the biology of some species [26], especially for endangered species, as any interference can have drastic impacts on viability. Most of the models developed for *Maculinea* species (Appendix S1) aimed at getting a better insight into their biology, especially the impact of the interaction with ants on population dynamics [23], [27], [28]. Applied management options were examined only by some models, either implicitly (via the impact of plant distribution [29], or by changing the number of host plants and ants [30]) or explicitly (by modelling the impact of certain management actions [25], [28]). Nevertheless, nearly all these studies focused on single and hypothetical populations. Only two models [25], [31] considered several patches, but the first one did not include any dispersal, while the second one considered a hypothetical 10 patch system with distances between them assumed to be short enough so that the dispersal rates are equal for all the patches. To our best knowledge, no model on a *Maculinea* species has ever included a spatially explicit description of a real landscape.

For long, *Maculinea* butterflies were considered to be extremely sedentary [15], [32]. This may explain why dispersal did not receive much attention in population models. However, increasing evidence is accumulating on their ability to move over distances similar to inter-patch distances in real landscapes (*M. alcon*: 500 m observed by capture-recapture and 2000 m suggested by data on colonization events [15]; all five *Maculinea* species: >2000 m [33]; *M. nausithous*: 3800 m [34]). This suggests that dispersal may be of crucial importance for *Maculinea* population viability, as it is for many species [35].

In this context, we developed a spatially explicit PVA model for *M. alcon* in the Northern region of the Netherlands. Applied conservation measures being usually taken at a local scale [5], [28], we distinguished four habitat patch networks within the region that differed in terms of number of habitat patches, their quality, connectivity, and occupancy rate (Table 1). We used these four patch networks to explore the relative effectiveness of the following four basic management options to enhance population viability: (i) enlargement of existing habitat patches, (ii) improvement of habitat quality, (iii) increasing connectivity by creation of new stepping stone habitat patches, and (iv) raising population size by reintroduction of reared butterflies. Management options were designed to incur the same costs, assuming a fixed amount of money is available to implement conservation measures. Our results demonstrate that the ranking of management options may considerably differ depending on the actual network configuration. Recommendations ensuing from the study can be readily used in the implementation of conservation and restoration actions (see [5]).

**Table 1:** Basic features of the four habitat patch networks selected within the study region for scenario analysis (Fig. 1).

Patch network	Number of patches	% occupied patches	Connectivity (%)*	Mean patch carrying capacity (ind.)	Mean patch area (ha)
Ballooërveld	7	0.71	0.24	52	0.780
Delleburen	9	0.33	0.16	26	0.599
Drents-Friese Wold	17	0.35	0.35	31	0.516
Dwingeloo	9	0.78	3.01	144	2.203

* Connectivity was calculated by dividing the suitable area (sum of the area of all patches) by the total area of the network (delineation of the minimum convex polygon around all the network patches).

## Methods

### Study Species and Region

In the Netherlands, *M. alcon* fly in July - early August and use two hosts to complete their life cycle [18]. After two to three weeks on their host plant Marsh Gentian (*Gentiana pneumonanthe*), on the flower buds of which females deposit eggs, caterpillars emerge and fall to the ground. Caterpillars may then be picked up by various *Myrmica* species but survive only in the nests of proper host species (in the case of *M. alcon* in the Netherlands, they are primarily *Myrmica ruginodis*, but also *M. scabrinodis*), in whose nests they overwinter and pupate.

**Figure 1:**
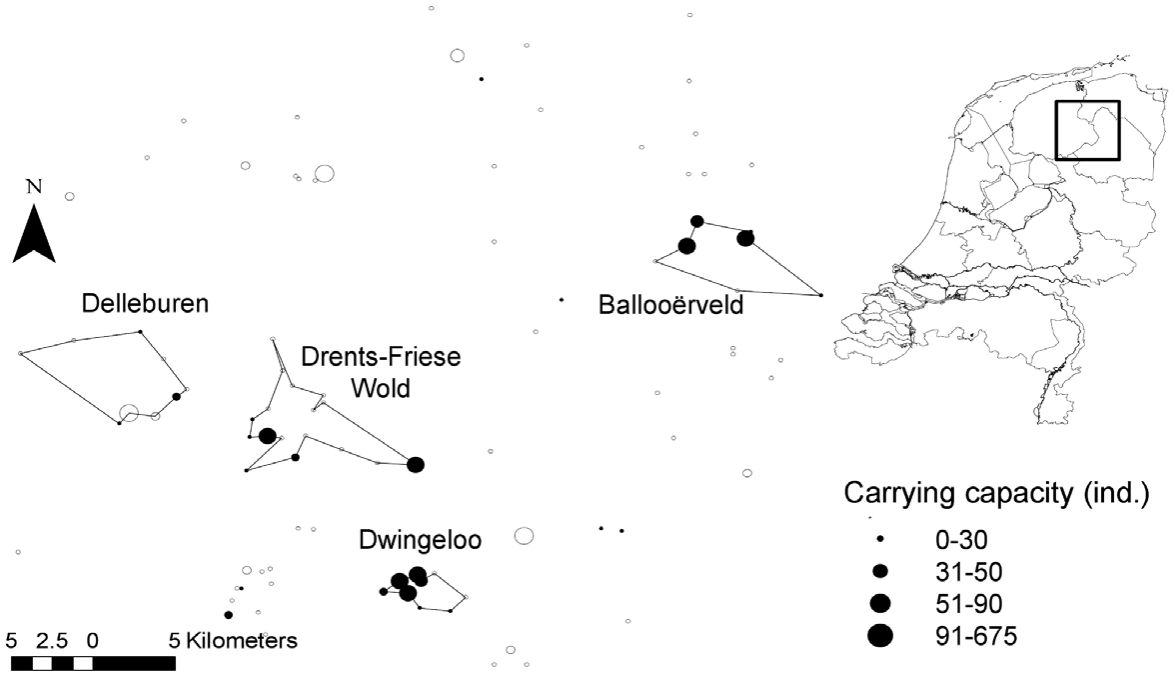
Map of *M. alcon* populations in the Drenthe-Friesland region, with the four selected habitat patch networks. Size of the symbol indicates the carrying capacity of the patch with closed circles used for occupied and open ones for empty habitat patches. Inset shows the map of the Netherlands with the study region delineated by the square.

**Figure 2:**
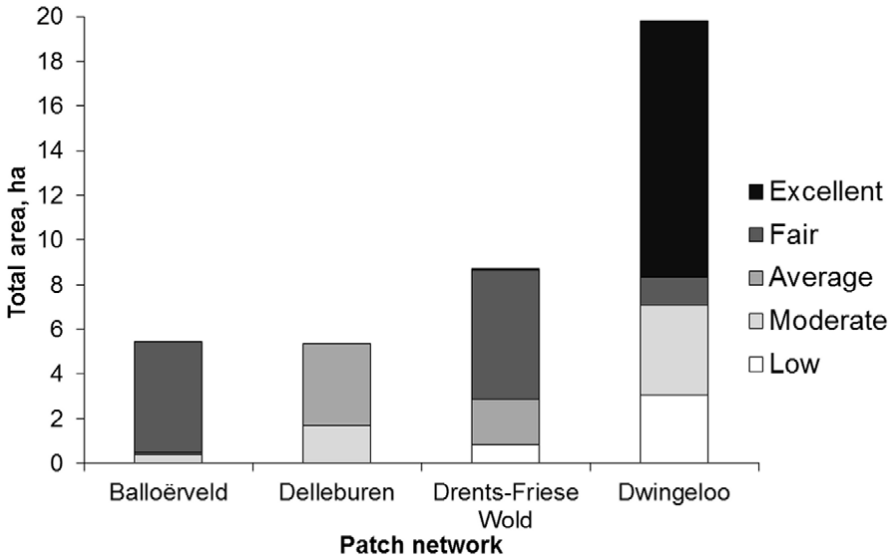
The four habitat patch networks differed in total area and quality of habitat patches. For each network the total area of the patches in each habitat quality category (low, moderate, average, fair and high) is shown.

Our study system is located in Drenthe-Friesland region, in the Northern part of The Netherlands (Fig. 1). The regional habitat network consists of 96 heathland patches with areas ranging from 0.005 ha to 7.5 ha (25^th^ percentile = 0.023 ha, median = 0.1 ha and 75^th^ percentile = 0.5 ha) and between-patch distances ranging from 282 m to 75 km. Within the region, we selected as targets for conservation measures four patch networks, differing in basic features such as number of habitat patches, their quality, connectivity, and occupancy rate (Table 1, Fig. 2).

### Model Parameterization

The model is a Structured Population Model, combining local population processes (the dynamics within each single population) with metapopulation processes via the dispersal between patches ([6] and references therein). It was developed with RAMAS/GIS software version 5.0 [36], using 1000 replications of each run and a time horizon of 200 years (200 generations). In order to keep the number of parameters reasonable, we did not explicitly model population dynamics of the plant and ant hosts; their impact on butterfly population was integrated in the carrying capacity of the habitat patches.

#### Local demography

Density-dependent processes including contest competition during the larval stages of *M. alcon* on the host plant and in the ant hosts nest are well-documented [10], [21], [28]. In our model, we modelled it with the Beverton-Holt equation:
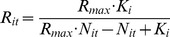
(1)with *R_it_* the growth rate of population *i* from year *t* to year *t+1*, *R_max_* the maximum growth rate, *N_it_* the population size of population *i* the year *t*, and *K_i_* the carrying capacity of patch *i*.

Carrying capacity of habitat patches was estimated based on five habitat quality categories defined by [18] on the basis of a set of patch descriptors (e.g. density of host plants, presence of host ants): low, moderate, average, fair and excellent, with *K* = 10, 30, 50, 70, and 90 individuals/ha, respectively.

*R_max_* was fixed at 3.29, according to estimates from Polish (3–3.3: [33]) and Italian populations (3.29: [16]). Egg count data from Dutch populations were available for the period 1993–2009. The large sampling variation associated with the small size of sampling plots (mostly 100 m^2^) precluded direct estimation of *R_max_* from these data. However, converted into adult counts by assuming each female lays 80 eggs with a 1∶1 sex ratio [15], [28], these egg counts were used to check whether the value of 3.29 was adequate for Dutch populations. To do so, we assessed the match between the *R_it_* values obtained using equation 1 and values observed for each habitat patch and year. No systematic over- or underestimation was found in predictions from equation 1 compared to observed *R_it_* values (paired t-test: t_211_ = 0.04, p = 0.97), and only 3.7% of observed growth rates were higher than 3.29. Equation 1 (both its structure and parameter values) was therefore judged adequate to estimate local demography for the populations modelled in this study.

Environmental stochasticity, the standard deviation of the series of residuals between observed and predicted (with equation 1) growth rates, was estimated at 1.12. It was implemented in the PVA model via the standard deviation of the population growth rate *R_t_*. Demographic stochasticity was included by sampling the number of individuals for each new generation from binomial distribution [36].

#### Correlation of demography between local populations

Correlation between the dynamics of local populations was estimated by Spearman’s correlation coefficients between time series of population growth rates, as estimated from egg counts. The mean correlation between two populations was 0.08. Only 4% (13 population pairs out of 325) were significant at the 0.05 level. Exactly 4% of significant correlations were expected under the null hypothesis of no correlation, as computed with a permutation test to take into account the non-independence of the data. Nowicki et al. (2007) [37] also concluded that there is no correlation between population dynamics for Polish population of the species. Consequently, no correlation of local population dynamics was included in the model.

#### Dispersal

Dispersal in metapopulations is usually estimated from Capture-Mark-Recapture (CMR) data or genetic data (F_st_). Unfortunately, none was available for *M. alcon* in the study area. F_st_ values were available for seven Danish populations (D. Nash, personal communication), but no estimate of population size exists to convert these into dispersal estimates. We therefore estimated dispersal by fitting the Virtual Migration model (VM: [38]) to data on 19 colonization events (incidental butterfly records within a surveyed area from the nearest existing population) in the Netherlands. VM models dispersal according to the following equations (for more details see [38]):
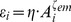
(2)

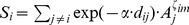
(3)

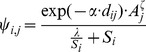
(4)with *ε_i_* the probability of emigration from the patch *i*, *η* the emigration probability from a patch of unit size, *ζ_em_* scaling the dependence of emigration rate on patch area *A*, *S_i_* the connectivity of patch *i*, *α* the distance-dependence in dispersal, *d_ij_* the distance between patches *i* and *j*, *ζ_im_* scaling the dependence of immigration rate on patch area, *ψ_i,j_* the probability of an individual leaving patch *i* to reach patch *j,* and *λ* scaling mortality during dispersal.

Starting from a synthesis integrating VM parameter values available for other *Maculinea* species [34], [39] and expert opinion (P. Nowicki, Jagiellonian University, Kraków, Poland), we used the vmsim module of VM to search for parameters best predicting the observed colonization pattern. Those were: *η* = 0.1, *ζ_em_* = –0.9, *α* = 2.1, *λ* = 0.05, *ζ_im_* = 0.75, predicting a distribution of successful dispersal events not significantly different from the one observed on colonization data (Kolmogorov-Smirnov: D = 0.38, p = 0.09). VM equations with these parameter values were used to calculate the proportions of individuals moving from one patch to each other patch in the system.

#### Initialization

27 out of the 96 patches in the network were occupied in 2003, used as year 1 in the model. Their initial population sizes were calculated from egg counts measured on the field in that year (assuming 80 eggs per female [28]). Seven populations had no record for numbers of individuals but presence of butterflies was recorded; their initial population sizes were estimated using a regression linking numbers of observed eggs and gentians (M.F. WallisDeVries: unpublished field census data from 2003): *N_eggs_*  = 2.0129•*N_gent_* +529.81 (R^2^ = 0.52, F_1,28_ = 28.7, p<0.0001).

### Model Analysis

#### Validation

The model was validated by comparing the mean occupancy times (proportion of years the patch was occupied) predicted by the model to field observations on the persistence/extinction of populations in these patches, made in 2010 (M.F. WallisDeVries: unpublished field census data), seven years after the situation used to initialize the model. Model predictions nicely matched the empirical data: the 16 patches observed to be occupied had a largely superior (t_23_ =  −2.41, p = 0.02) average predicted occupancy time (91 years) compared to seven patches in which populations went extinct (6 years).

#### Sensitivity analysis

Local sensitivity analysis was conducted separately for the whole region and for each network by altering the following parameters in the baseline scenario model: carrying capacity, maximum growth rate, initial abundances and dispersal rates. As a response variable we used the population size (total abundance in the system) associated to a 50% quasi-extinction risk. The quasi-extinction risk is a measure of viability quantifying the probability that (the system of) population(s) will fall below a threshold population size at least once during the simulation time period [36], [40]; it is basically an extension of the notion of extinction risk to population size thresholds above 0. The value of each parameter was changed by ±5% and the sensitivity coefficient was calculated using the following equation [41], [42]:
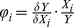
(5)


with *δY* the change in the response variable*Y* (here the population threshold below which the system has 50% chance to fall at least once during the simulated 200 years), *δX_i_* the change in the model parameter *X_i_*, and 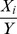 a normalizing coefficient used to remove the effect of the units. The sensitivity index of each parameter was computed as the average of the absolute values of *ϕ* sensitivity coefficients [42] for both +5% and -5% changes.

To test the impact of adding some between-population correlation, we calculated expected correlation coefficients between all possible pairs of populations using the following equation:
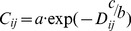
(6)predicting the correlation coefficient *C_ij_* between populations *i* and *j* according to the distance *D_ij_* between the two populations, and *a*, *b*, and *c* the function parameters [36]. We ran a model with two different levels of correlation between the population growth rates: (1) *a* = 0.91, *b* = 1 and *c* = 3, resulting in a correlation of 0.91 at 0 km, 0.33 at 1 km, 0 at 2 km and (2) *a* = 0.8, *b* = 1 and *c* = 0.1, resulting in a correlation of 0.8 at 0 km, 0.29 at 1 km, 0.23 at 10 km and 0.18 at 50 km.

#### Management options

The relative impact of four alternative management options was compared: (1) *habitat enlargement*, (2) *habitat quality improvement*, (3) *stepping stones creation*, and (4) *reintroduction* of reared butterflies. These management options were designed in a way to incur an approximately equal cost of 20,000 € to allow comparison in terms of cost/benefit ratio. They were implemented for each network separately to assess if different networks required different management measures in order to efficiently preserve the species. Table 2 provides details for the four options: management actions undertaken, costs, practical implementation in the model, and time lag before they affect the patch network.

**Table 2:** Specification of the management options tested.

Management option	Management action		Management cost	Implementation in the model	Time lag of effect
**Habitat enlargement**	From adjoining degraded wet heathland/forest: sod-cutting, liming, removal of trees and prevention of drainage (filling ditches)		5,000 €/ha ^1^	Existing patches were enlarged to 0.5 ha when smaller (viable populations are sustained by >0.5 ha for *M. alcon* ^2^). When more than 4 ha (20,000 €) would be needed to enlarge all existing patches, preference was given to occupied patches. Final habitat quality was set to average.	Habitat quality is increasing linearly over 20 years before the target *K* is achieved ^2^
**Habitat quality improvement**	Small-scale sod-cutting + liming, sowing of gentian seed, improving hydrology, adjusted grazing or mowing, controlling tree establishment		2,500 €/ha for 1 quality category up, irrespectively of the current habitat quality category ^1^	Patches with habitat quality lower than a certain threshold category (Ballooërveld and Drents-Friese Wold: excellent; Delleburen: fair; Dwingeloo: average), defined according to the initial habitat quality within each network and the 20,000 € budget limit.	A linear increase in *K* during the next 5 (one category up), 7 (2 categories up), 10 (3 categories up), or 12 years (4 categories up) ^2^
**Stepping stones creation**	From agricultural land: topsoil removal of 50 cm and introduction of cut heather with gentian seed.	From degraded wet heathland/forest: sod-cutting, liming, removal of trees and prevention of drainage (filling ditches)	15,000 €/ha from agricultural land ^1^ and 5,000 €/ha from degraded wet heathland/forest ^1^	A series of 0.5 ha average habitat quality patches were created in the landscape matrix within each network. For each new patch to be created, the current land use at its exact position was determined. The number of stepping stones (4–6) differed between the networks as a function of the cost difference between the restoration of patches from agricultural (15,000 €/ha) and degraded heathland/forest areas (5,000 €/ha). Stepping stones were placed in poorly connected locations (inter-patch distance >2 km maximum dispersal distance ^3^) in order to connect an occupied patch with a vacant patch of sufficient carrying capacity (wherever possible). The distance to each of the two patches the stepping stone aimed to connect was less than 1.5 km (0.25–1.3 km with a similar c. 0.7 km average for the four networks).	A linear increase in *K* during the next 20 years, preceded (agricultural land only) by a 10 yr lag ^2,4^
**Reintroduction**	A total of 14 captive-reared individuals were reintroduced into vacant patches of each network during 8 years		2,300 € for 14 butterflies per network per year	A minimum of four individuals were introduced in a given patch, with higher priority given to the patches presenting a higher carrying capacity.	

1 Rob van der Burg (Bosgroep-Zuid) and René Gerats (Stichting Het Limburgs Landschap, personal communication); www.groenblauwediensten.nl.

2 WallisDeVries 2004 [18].

3 Maes et al. 2004 [15].

4 WallisDeVries unpublished data.

## Results

### Baseline Scenario

**Figure 3:**
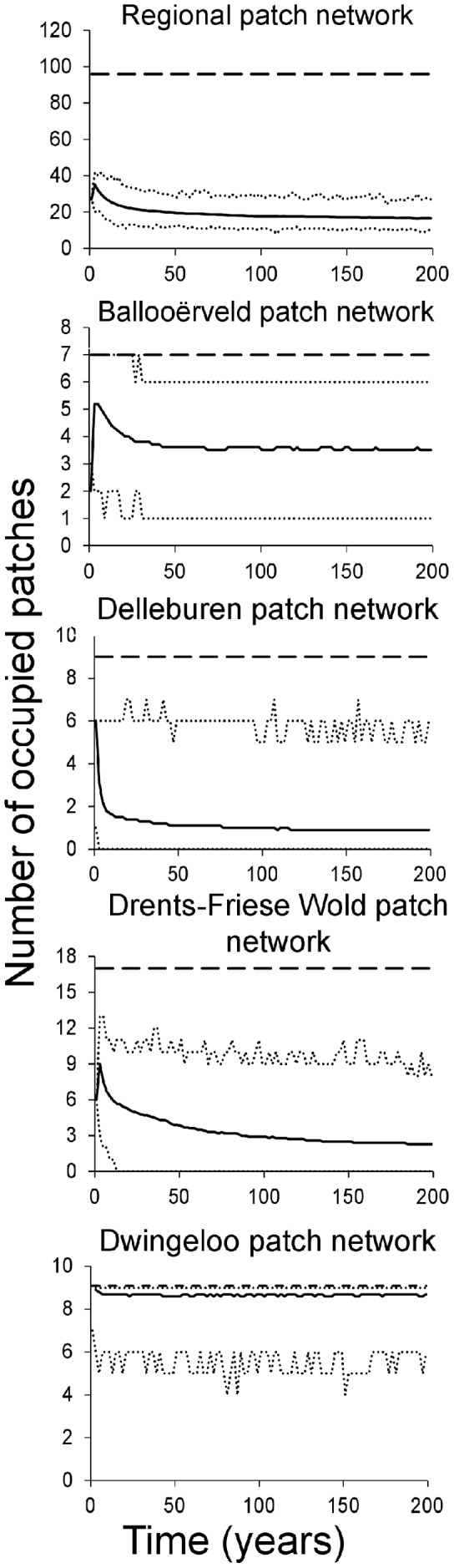
Population occupancy over the simulated period (200 years) for each habitat patch network under the baseline scenario. Solid line: average number of occupied patches; dotted lines: 95% confidence limits for 1000 replications; dashed line: total number of patches in the system.

The model predicted a decline of population size in the region over the next 200 years, with only 17 patches being occupied at the end of the 200 yr period (to compare to 27 occupied in 2003, starting point of the simulation, Fig. 3). Viability of the four networks differed greatly: Dwingeloo > Ballooërveld > Drents-Friese Wold > Delleburen. Delleburen was highly vulnerable, presenting a 78% extinction probability under the baseline scenario. Only the Dwingeloo network was predicted to keep a stable (and high) occupancy rate. This ranking coincides with the ranking according to three basic features known to affect population viability (carrying capacity, occupancy and connectivity: Table 1, Fig. 2). Nevertheless, the total number of populations is decreasing, illustrating the threat existing for the species in the Northern part of The Netherlands.

### Sensitivity Analysis

**Figure 4:**
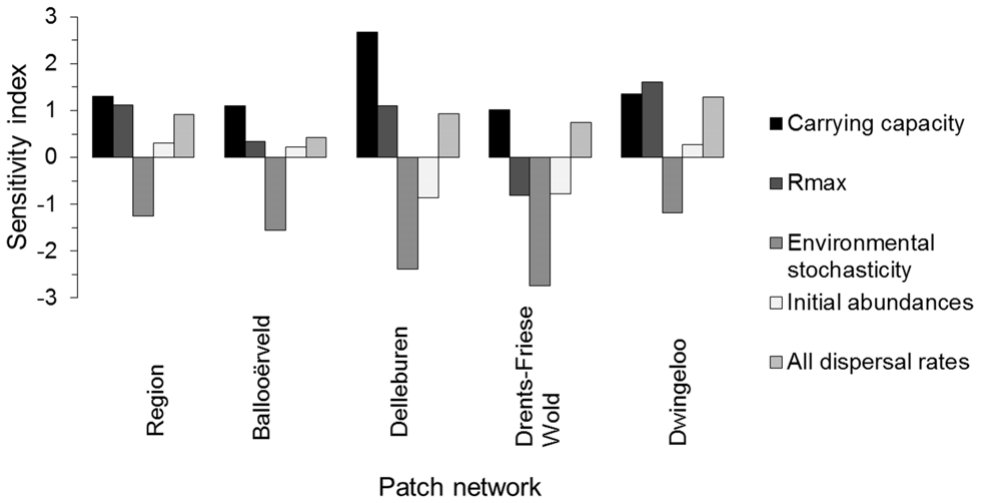
Sensitivity indices of model parameters, as quantified at regional and individual networks levels. A higher absolute value of the index means that model predictions were undergoing a larger change for a given change in the model parameter ([42]). The direction of the impact (+ or – sign), indicates a positive and a negative effect on viability of an increase in the model parameter, respectively.

Sensitivity analysis (Fig. 4) revealed that model predictions, both at regional and individual network levels, were highly sensitive to carrying capacity *K* and environmental stochasticity, moderately sensitive to maximum growth rate *R_max_* and dispersal rates, and fairly insensitive to initial abundances. Viability was improved by a higher *K*, a higher *R_max_* (except for Drents-Friese Wold), a lower magnitude of environmental stochasticity and higher dispersal rates among the habitat patches. Nevertheless, these general trends vary among the networks (Fig. 4), with Ballooërveld and Delleburen being sensitive to carrying capacity and environmental stochasticity (but with a quite larger impact in Delleburen), Drents-Friese Wold being especially sensitive to environmental stochasticity, and Dwingeloo being relatively equally sensitive to all parameters (except initial population sizes).

The inclusion of between-population correlation decreased viability: compared to the baseline model, the population size corresponding to a 50% quasi-extinction risk was lower by 2% and 25% for the two levels that were tested, respectively. However, the second level corresponds to a correlation affecting a very large spatial extent, and seems unlikely under current conditions.

### Management Options

**Figure 5:**
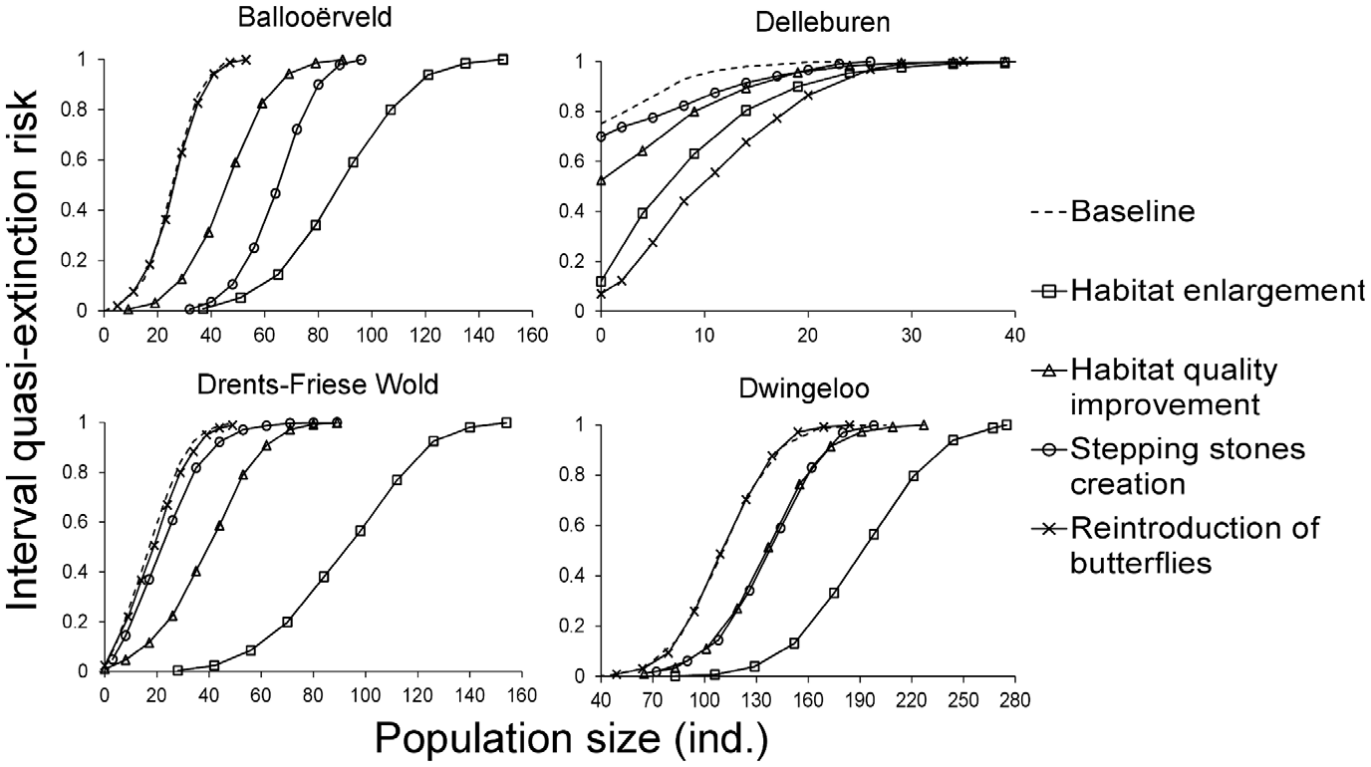
Assessment of *M. alcon* viability in the four habitat patch networks under the baseline and management scenarios . The quasi-extinction risk is a measure of viability quantifying the probability that (the system of) population(s) will fall below a threshold population size at least once during the simulation time period [36], [40]. This risk is given here for the full range of population size thresholds, from a 0% to a 100% risk of falling below the threshold; for a given population size, a lower quasi-extinction risk means a higher viability. Habitat enlargement is the best management option in three patch networks (Ballooërveld, Drents-Friese Wold and Dwingeloo), and reintroduction is the best for Delleburen network.

The comparison of four management options with similar financial costs revealed that investment in *habitat enlargement* was the single most cost-effective technique to improve the overall viability of the species in the region (Fig. 5). However, the impact of the other management options differed between networks, in a way that was clearly linked to the network features (Table 1, Fig. 2).

*Habitat quality improvement* resulted in an increase of viability that was less than half the one gained from *habitat enlargement*. There are two reasons for that. The first one is that we assumed a similar cost for restoring from degraded heathland (*enlargement*) and from low quality habitat (*improvement*), creating in both cases average quality habitat; the gain in *K* is 50 individuals/ha for *enlargement*, but only 40 individuals/ha for *improvement*. More importantly, *habitat quality improvement* is inefficient in many patches because of their tiny size, which keeps their *K* very low even when habitat quality is high. For a similar cost, *habitat enlargement* allows increasing the size of some patches (and hence *K*) to a non-negligible value, able to support a viable population.

*Stepping stones creation* was efficient only in Ballooërveld, and to a lesser extent in Dwingeloo. Contrary to Delleburen and Drents-Friese Wold, these two networks were characterized by a small number of patches with a high occupancy rate; therefore, newly created habitat patches are likely to be rapidly colonized by dispersal events. Stepping stone populations improve viability through (1) an increased buffer effect against extinction typical for a metapopulation with more patches, and (2) an enhanced connectivity, clearly exemplified by the poorly connected Ballooërveld network.

The *reintroduction* of butterflies reared in captivity had a negligible impact on viability in three out of the four networks, whereas it was the best option in Delleburen. This network was characterised by poorly connected habitat patches with a high proportion of vacant patches, some of them with rather high *K* (Fig. 1). In such a case, reintroduction allows to maintain/recreate populations in several patches, thereby strengthening the metapopulation buffer effect against extinction.

## Discussion

To our best knowledge, our model is the first spatially explicit PVA for a *Maculinea* species, integrating both a real multipatch network and a quantitative description of the magnitude of between-patch dispersal. Furthermore, it is also the first of such models to be financially explicit. Initialised with the 2003 situation, the model predicted an occupancy pattern closely matching observed occupancy in the field in 2010, and it can therefore be considered as a validated and reliable tool in the context of predicting population viability and comparing management scenarios, in order to develop conservation guidelines for the species in the Northern Netherlands.

Altogether, the future of *M. alcon* in the Northern region of the Netherlands is far from safe. The number of populations is dangerously small and is predicted to further decline over the next decades if the current conditions prevail. The probabilities are high that the populations reach very low abundances: 50% probability that the total abundance in the whole region would decline to 262 individuals only in 200 years. Extinction risk may be even larger in reality than predicted here because of processes adversely affecting very small populations (such as genetic inbreeding or Allee effect [43]) that were not included in our model. Management actions are clearly necessary to improve the conservation status of the species.

Viability of *M. alcon* was highly sensitive to parameters affecting the local demography: carrying capacity, environmental stochasticity, and to a lower extent maximum growth rate. Local demography has also been found a very important aspect of metapopulation viability for several butterfly species (e.g. *Icaricia icarioides fenderi* [44], *Euphydryas aurinia* [45], *Boloria eunomia* [46], *Boloria aquilonaris* [47]). Similarly, previous models on *Maculinea* species focussed mostly on the comparison of different habitat restoration options to improve habitat patch quality: e.g. burning the vegetation layer every 4–6 years [28], using small-scale sod cutting [18], or mowing with different frequency and timing [25]. This focus on habitat quality is important as it is the one direct measure likely to increase the carrying capacity of habitat patches.

However, by implementing dispersal in a spatially explicit quantitative way and by ranking management options that were controlled to be equal in their financial cost, our model was able to bring important and new conclusions, demonstrating that (1) improving quality of existing habitat patches, though beneficial, was not the best option, and that (2) the different patch networks showed contrasted predicted impact of the management options.

Overall, the best management option with 20,000 € at hand would be to restore 4 ha of degraded heathland to enlarge existing habitat patches. It outperforms the improvement of habitat quality of existing patches for two reasons. (1) For the same cost (restoration from degraded heathland), it brings a larger increase in carrying capacity (+50 ind./ha instead of +40 ind./ha), but this might partly be due to an approximation of the cost quantification for habitat quality improvement. (2) More importantly, even after their quality is improved by restoration, many patches are too small to sustain a local population viable on the long term. Only their enlargement to the required minimum area [18] is able to increase their carrying capacity to the threshold needed for such population viability. Unfortunately, this is a common situation in intensively used and highly fragmented areas such as the Netherlands, responsible for the poor state of *M. alcon* populations.

**Figure 6:**
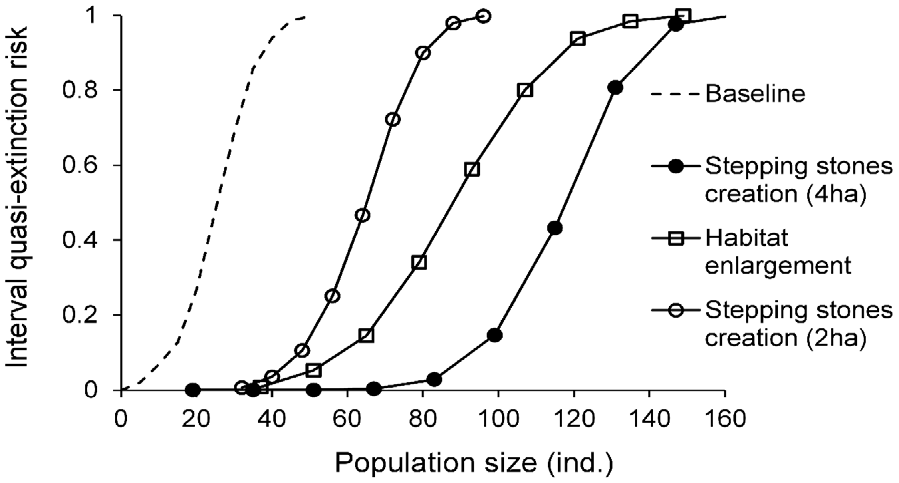
Restoration cost from agricultural land determines the best placement of restored area. In Ballooërveld patch network, the ranking of *stepping stone* vs *habitat enlargement* management options depends on the relative restoration cost: if the same surface can be restored, viability is improved more by placing restored area as stepping stones (*stepping stone creation 4* *ha* scenario); but the doubled cost of restoration from agricultural land makes *habitat enlargement* the best management option under the equal cost allocation approach.

The creation of new stepping stone habitat patches was never the best management option. However, concluding that connectivity is currently sufficient would be misleading, especially in the case of the poorly connected Ballooërveld patch network. Indeed, the lower impact of restoring habitat area in the form of new stepping stone patches than in the form of enlarged existent patches is due to the high price of restoring habitat from agricultural land, doubled compared to restoration from degraded heathland. Therefore, with the same money for conservation, the area that can be restored under the stepping stone creation scenario was only 2 ha (vs 4 ha for patch enlargement). If a fixed 4 ha area can be restored, placing it in stepping stone patches would lead to a higher improvement of species viability (Fig. 6), confirming the biological importance of limited connectivity in determining viability of *M. alcon* in that network. Additionally it underlines how failing to consider the costs associated to each management option can lead to an opposite conclusion about the best practically feasible management option.

The viability predictions were also sensitive to the magnitude of between-patch dispersal, especially in the Dwingeloo network, the network where the extinction risk is not as high as in the other three networks. This, together with the previous conclusion that creating stepping stone patches would largely improve population viability, emphasizes the role of dispersal in the long-term persistence of the species. Consequently, this underlines the importance to explicitly include habitat configuration and dispersal in PVA models for *M. alcon*, and therefore to obtain precise estimates of dispersal for the species. Considering all the accumulated knowledge on different aspects of the biology and ecology of *Maculinea* species across Europe (e.g. [16], [21], [48], [49], [50]) the global paucity and limited quality of available dispersal estimates surprised us. Published dispersal kernel estimates [39] failed to predict dispersal to distances that are travelled by butterflies in the field, as judged, for example, by records on colonization events up to 7 km in The Netherlands (those used to parameterize dispersal in our model). One clear reason is that estimates of the dispersal for this species are biased due to the small scale of the study areas (maximum inter-patch distance in these studies was 450 m [51], 650 m [15], 1200 m [39], and 5800 m [34]); this is a known general limitation of Capture-Mark-Recapture data to estimate dispersal [52]. Dispersal seems to be a clear gap in the knowledge on the species, which should be resolved as soon as possible by further research. A relatively easy and fast way to estimate dispersal would be to use genetic data, which is further facilitated by the availability of microsatellite markers for this species [53].

Reintroduction was suggested by Maes and colleagues [15] as an additional management option, which should be exploited cautiously together with an enhancement in carrying capacity to increase population viability of *M. alcon*. Our results showed that reintroduction was usually predicted to have a limited impact on population viability of *M. alcon* in the region studied. Only in the case of the Delleburen patch network, which scored the worst against all features considered (Table 1), viability was improved by reintroduction of captive-reared butterflies.

We focused on four alternative management options; however the costs needed to implement them might differ for *M. alcon* inhabiting other habitat types (meadow instead of heathland) or for restoration from other land use types (enlarging the habitat patch using arable lands would be more expensive than from wet heathlands, as envisioned here). Moreover, diverse combinations of the explored management options with partial allocation of money to each of them is possible in order to achieve the most effective management strategy ensuring increased population viability yet at least cost. Our model can be readily used by local managers in order to test possible combinations to identify the most cost-efficient management strategy for each particular region.

Many studies (e.g. [54], [55], [56]) explored the impacts of habitat quality and fragmentation on population viability by simulating artificial landscapes offering the desired combination of the studied factors. This is to counteract the obvious peculiarity of real ecological systems: study situations must be taken as they are offered, usually far from the well-designed laboratory experiments. In this study, we took advantage of the availability of four real patch networks, contrasting in their main local and regional features (Table 1) to explore how these features influence the impact of management options. Our results clearly indicate that these networks differed both in their sensitivities to model parameters and in the ranking of financially equivalent management options. In this specific case of *M. alcon* in the Northern Netherlands, our predictions stress the cost effectiveness of enlarging existing habitat patches to improve species viability, whereas creating new stepping stone patches in the middle of the agricultural matrix might do better but at a doubled cost. We conclude by arguing that the conservation guidelines would have been very different if the PVA model had failed to integrate both (1) a spatially explicit description of the landscape and the dispersal of the species, and (2) a financial quantification of the management options, to make their comparison on a realistic basis. We encourage researchers to take these two aspects into account whenever possible.

## Supporting Information

Appendix S1 **Review of the population dynamics models developed for** ***Maculinea*** **species so far.**
(DOC)

## Acknowledgments

This study would not have been possible without the sustained effort of numerous volunteers to monitor *Maculinea* populations; we are especially grateful to J. de Vries who coordinated the monitoring in Drenthe. We would like to thank P. Nowicki (Jagiellonian University, Kraków) for fruitful discussions and inputs on parameterizing the dispersal part of the model, and H. De Leeuw and G. Oostermeijer (University of Amsterdam) for preliminary work. This paper is contribution BRC260 of the Biodiversity Research Centre at UCL.
