# Supplementary material for: Spatially and Financially Explicit Population Viability Analysis of Maculinea alcon in The Netherlands
Source: PLoS One. 2012 Jun 14;7(6):e38684. doi: 10.1371/journal.pone.0038684 (PMC3375285; doi:10.1371/journal.pone.0038684)
Supplement: Appendix S1 — Review of the population dynamics models developed for Maculinea species so far. (DOC) [file pone.0038684.s001.doc]

Supporting Information

Appendix S1.

Review of the population dynamics models developed for *Maculinea* species so far (to our best knowledge)^*^

| Paper | Butterfly species | Location | Model purpose | Plant host dynamics | Ant host dynamics | Number of patches (total area) | Dispersal | Environmental stochasticity |
| --- | --- | --- | --- | --- | --- | --- | --- | --- |
| [Hochberg, Thomas & Elmes 1992](#_ENREF_5) | *M. rebeli* | Spain | Better understanding of species biology | No | No | 1 patch | No | No |
| [Hochbe*rg et a*l. 1994](#_ENREF_4) | *M. rebeli* | Spain, France | Exploration of species biology and predicting minimal requirements for population persistence | No | Yes | 1 patch | No | No |
| [Clar*ke et a*l. 1998](#_ENREF_1) | *M. rebeli* | Spain, France | Exploitation of potential impact of habitat quality on population viability | Implicit | Yes | 1 patch | No | No |
| [Griebeler & Seitz 2002](#_ENREF_3) | *M. arion* | Germany | Exploration of species biology and estimation of management options | No | No | 8 closed populations (25 ha) | No | No |
| [Mouqu*et et a*l. 2005a](#_ENREF_8) | *M. alcon* | France, Spain, Poland | Exploration of the conservation measures for population preservation | Yes | Yes | 1 patch | No | No |
| [Mouqu*et et a*l. 2005b](#_ENREF_9) | *M. arion* | United Kingdom, France, Sweden | Evaluation of how endogenous and exogenous factors both impact population dynamics | No | No | 1 patch | No | No |
| [Joh*st et a*l. 2006](#_ENREF_7) | *M. nausithous*, *M, teleius* | France, Poland | Assessment of different mowing regimes for butterfly population preservation | No | No | 10 patches (area not mentioned) | Yes, simplified | No |
| [Griebeler 2011](#_ENREF_2) | *M. arion* | Germany | Comparing a deterministic mathematical model to an individual based model | No | No | 1 patch | No | No |

^*^The table lists papers simulating to a certain extent the population dynamics of *Maculinea* species; other models exploiting life history of the species but not focussing on butterfly population dynamics (e.g. [Hovesta*dt et a*l. 2007](#_ENREF_6)) are not included

**LITERATURE CITED**

Clarke, R., Thomas, J.A., Elmes, G.W., Wardlaw, J.C., Munguira, M.L. & Hochberg, M.E. (1998) Population modelling of the spatial interactions between *Maculinea rebeli*, their initial foodplant *Gentiana cruciata* and *Myrmica* ants within a site. *Journal of Insect Conservation,* **2,** 29-37.

Griebeler, E.M. (2011) Are individual based models a suitable approach to estimate population vulnerability? - a case study. *Computational Ecology and Software,* **1,** 14-24.

Griebeler, E.M. & Seitz, A. (2002) An individual based model for the conservation of the endangered Large Blue Butterfly, *Maculinea arion* (Lepidoptera : Lycaenidae). *Ecological modelling,* **156,** 43-60.

Hochberg, M.E., Clarke, R.T., Elmes, G.W. & Thomas, J.A. (1994) Population-Dynamic Consequences of Direct and Indirect Interactions Involving a Large Blue Butterfly and Its Plant and Red Ant Hosts. *Journal of Animal Ecology,* **63,** 375-391.

Hochberg, M.E., Thomas, J.A. & Elmes, G.W. (1992) A Modeling Study of the Population-Dynamics of a Large Blue Butterfly, *Maculinea rebeli*, a Parasite of Red Ant Nests. *Journal of Animal Ecology,* **61,** 397-409.

Hovestadt, T., Mitesser, O., Elmes, G.W., Thomas, J.A. & Hochberg, M.E. (2007) An evolutionarily stable strategy model for the evolution of dimorphic development in the butterfly *Maculinea rebeli*, a social parasite of *Myrmica* ant colonies. *American Naturalist,* **169,** 466-480.

Johst, K., Drechsler, M., Thomas, J. & Settele, J. (2006) Influence of mowing on the persistence of two endangered large blue butterfly species. *Journal of Applied Ecology,* **43,** 333-342.

Mouquet, N., Belrose, V., Thomas, J.A., Elmes, G.W., Clarke, R.T. & Hochberg, M.E. (2005a) Conserving community modules: A case study of the endangered lycaenid butterfly *Maculinea alcon*. *Ecology,* **86,** 3160-3173.

Mouquet, N., Thomas, J.A., Elmes, G.W., Clarke, R.T. & Hochberg, M.E. (2005b) Population dynamics and conservation of a specialized predator: A case study of *Maculinea arion*. *Ecological Monographs,* **75,** 525-542.
